# Laplacian Scores-Based Feature Reduction in IoT Systems for Agricultural Monitoring and Decision-Making Support

**DOI:** 10.3390/s20185107

**Published:** 2020-09-08

**Authors:** Giorgos Tsapparellas, Nanlin Jin, Xuewu Dai, Gerhard Fehringer

**Affiliations:** 1 Department of Maritime and Mechanical Engineering, Liverpool John Moores University, Liverpool L3 3AF, UK; g.tsapparellas@ljmu.ac.uk; 2 Department of Computer and Information Sciences, Northumbria University, Newcastle upon Tyne NE1 8ST, UK; gerhard.fehringer@northumbria.ac.uk; 3 Department of Mathematics, Physics and Electrical Engineering, Northumbria University, Newcastle upon Tyne NE1 8ST, UK; xuewu.dai@northumbria.ac.uk

**Keywords:** Laplacian scores, data reduction, sensors, Internet of Things (IoT), LoRaWAN

## Abstract

Internet of things (IoT) systems generate a large volume of data all the time. How to choose and transfer which data are essential for decision-making is a challenge. This is especially important for low-cost and low-power designs, for example Long-Range Wide-Area Network (LoRaWan)-based IoT systems, where data volume and frequency are constrained by the protocols. This paper presents an unsupervised learning approach using Laplacian scores to discover which types of sensors can be reduced, without compromising the decision-making. Here, a type of sensor is a feature. An IoT system is designed and implemented for a plant-monitoring scenario. We have collected data and carried out the Laplacian scores. The analytical results help choose the most important feature. A comparative study has shown that using fewer types of sensors, the accuracy of decision-making remains at a satisfactory level.

## 1. Introduction

The Internet of Things (IoT) interconnects and embeds objects, machines and devices, forming a highly distributed network of device broadcasting with humans and other devices [1]. Recent application areas progressing within the IoT sector include smart cities, agriculture, building, healthcare, and shopping [2,3,4]. This paper proposes an open-source [5] and low-cost Long-Range Wide-Area Network (LoRaWAN) solution for strawberry-plant monitoring.

Conducting data-mining on raw IoT data will help to reduce the cost of powering sensors, the amount of packet transmission, latency, and response delay [6]. Moreover, the discovery of information from raw data improves system performance. Data-mining generates knowledge models from data received to support decision-making. Common methods include data compression, data-mining, and data reduction [6].

Recent research introduces a range of methods, including compression [7] and reconstruction [8], aggregation [9], redundancy removal, and reduction of the number of sensors [6] using time-discounted histogram encoding. To replace multiple sensors that send appliance energy usage in households, smart phone data is used as the only data source to estimate user activities [10]. A lightweight monitoring framework has been developed to cope with limited processing capabilities. It adapts the amount of data disseminated through the network over time [11]. Another framework transmits updates when the sensor readings are detected to be unusual, and have triggered dissemination [12], adapting the monitoring sensing intensity and dynamically adjusting the data volume payload.

Reducing the amount of data to be analyzed in IoT systems can be done either offline or online. The offline analysis is to collect data as much as possible during trials, to conduct offline analysis, and discover patterns. During the real-time operation, data will be checked against such learned patterns while running lightweight data analysis programs, for example signature-based network-intrusion-detection systems [13]. The online analysis operates data reduction in real time, to calculate the difference from normal behaviors, for example anomaly-based detection network-intrusion-detection systems [11,12,14]. The offline approach might out-perform the online approach in finding the previously trained events or situations, but the second approach would be better if unknown situations happens during real-time operation. This paper focuses on the offline approach. Here, the feature selection will not serve as pre-processing techniques for data-mining only, but also determine the sources of data to be collected in deployment and operation.

The rest of this paper is structured as follows: Section 2 showcases Related Work, Section 3 shows the motivation and analysis behind the Data reduction in IoT monitoring, Section 4 presents the Problem and System Architecture, including the design and implementation of the proposed LoRaWAN-based IoT system for strawberry-plant monitoring, Section 5 shows the experimental results and analysis including experimental set-up and sensors calibration, traffic analysis, data visualization, feature selection and evaluation, and example in decision support, and Section 6 provides conclusions with any future research directions.

## 2. Related Work

### 2.1. Usage of Sensors in Agriculture

The usage of sensors and actuators has been replacing the traditional human-intensive ways of monitoring in agriculture [15]. Sensors can measure environmental parameters and convert them into meaningful signals [16], for example, water resource monitoring for irrigation [17]. It is reported that in 2000, there were approximately 525 million farms on record across the globe, but none connected to the IoT. However, by 2025 for the same base of 525 million farms, it is expected for there to be around 600 million sensors installed, connected and in use in these farms [18]. The technological advancement as well as size abatement of devices make employment of sensors feasible for agriculture applications [16].

### 2.2. IoT LPWAN Communication Protocols: LoRa and LoRaWAN

Low-power wide-area (LPWAN) communication protocols are designed for low-power consumption, suitable for applications which demand limited efforts for maintenance. One of the protocols, LoRaWAN, has been introduced by the LoRa Alliance organization as the protocol for low-power and wide-area coverage [19]. LoRaWAN, which stands for long-range wide-area network, defines the communication protocol and the system architecture for the network [20].

By definition, LoRa is the physical layer or the wireless modulation used to create long communication links. In terms of the LoRa functionality, an end-device communicates to a gateway which is employing LoRa with LoRaWAN. To be more specific, a LoRa gateway passes raw LoRaWAN packets from the end-devices to a network server [19]. Major advantages of LoRa are its low-power consumption, long-range capability, security and relatively easily expandable network. However, LoRa advantages have their trade-offs: for example, the time delay for the data to be stored in the cloud after being obtained, and the final data usage or display [21]. Therefore, it might not be the ideal choice for those applications requiring immediate responses or high-resolution data.

However for low-cost and low-power IoT systems, the data transmission is constrained. Therefore, how to reduce the volume of data to be sent from a LoRa node to a network server, while still enabling data-driven decision, is a challenge.

### 2.3. Feature Reduction

To reduce the number of features to be used, the main data-mining methods include: feature selection, which selects a subset of the original feature set; and feature extraction, which creates a set of new features by combining original features. The choice of selecting features are problem-dependent, but the resulting subset features should remain a faithful, perhaps simplified representation to the original data set and preserve the intrinsic knowledge accurately. This paper focuses on feature selection.

Feature selection methods were used to identify the set of features which brings high accuracy to detect cyber-attacks [22]. It has been found that features have discriminatory contribution to classification accuracy in identifying attacks. Some features are redundant, irrelevant, partially relevant to the learning target and some even reduce accuracy, for example noise.

In addition, feature construction or feature transformation can create new features or transform existing features into a new set of features, smaller than the original set [23]. This method requires decent domain related knowledge, for example the understanding of energy usage patterns as shown in [23]. Principal component analysis (PCA) also summarizes data into fewer dimensions by projecting it onto an orthogonal basis.

Deep learning has demonstrated high performance in terms of accuracy [24]. However in the setting of real-time operation in IoT, response time is one of key requirements and edge devices or even gateways have limited computational resources to use the computationally demanding method deep learning, especially for large scale of IoT systems. In addition, the results from deep learning is difficult to be interpreted. This method is especially unpractical when human involves in analysis, monitoring, decision-making and control.

## 3. Data Reduction in IoT Monitoring

To illustrate the feature reduction, we provide a sample scenario in a plant-monitoring context. For example, some sensors can be used: temperature w(t), humidity h(t), lighting b(t) and soil moist s(t). The decision-making for a specific action can be represented as a function $f:{ℜ}^{4k}\to ℜ$ as follows:

(1) d(t)=f(w(t),h(t),b(t),s(t),θ)

where d(t)∈ℜ is the decision variable representing the action to be taken. For example, d(t)=1 means watering and d(t)=0 means no watering. And $w\left(t\right)\in {ℜ}^{k}$, $h\left(t\right)\in {ℜ}^{k}$, $b\left(t\right)\in {ℜ}^{k}$ and $s\left(t\right)\in {ℜ}^{k}$ are data vectors for temperature, humidity, lighting and soil moist for the last *k* samples until time *t*, For example, $w\left(t\right)={\left[w\left(t-k+1\right),w\left(t-k+2\right),\cdots ,w\left(t-1\right),w\left(t\right)\right]}^{T}$ is the last *k* samples of the temperature at time *t*. *k* is referred to as the sampling window.

The research question is how to make the correct decision with less data. More specifically, the data reduction problem can be stated as follows: Are all these four types of data needed to make the decision? Would it be possible to just use three type of data and which three type of data should be selected to make the decision?

### 3.1. Feature Selection Using Laplacian Score

Carrying out data analysis on many features is always computationally expensive. Its computational complexity increases while the dimensions or the number of features increase. Therefore, to select the most important features becomes necessary, especially in source-limited situations.

There is a rich range of dimensionality reduction methods. Some are suitable for classification, for example, to rank features using neighborhood component analysis, to rank features using minimum redundancy maximum relevance algorithm, to estimate predictor importance for classification tree. Some are suitable for regression, to select those independent variables which have the best relation to the predictor, i.e., the dependent variable, for example, to rank features using F-test. This method will be useful if the dependent variable is known and its data is collected. In our IoT system, it has a set of sensors for monitoring, but its predicting variable is unknown. Therefore we will need to consider feature selection for unsupervised learning. For unsupervised learning, Laplacian scores have been used to rank features.

Laplacian score was designed to select features in unsupervised learning [25]. Feature selection in unsupervised learning is more difficult than supervised learning, due to lacking of class labels to guide search. Laplacian score was introduced as a filter method to evaluate a feature by “its power of locality preserving”, using local neighborhood relationships between data points [25].

For feature selection in supervised learning, Laplacian score has been used for multi-label classification, to measure feature relevance [26] to be used together with manifold learning which is non-linear dimensionality reduction [27]. For feature selection in unsupervised learning, Laplacian score concept has been used to produce pseudo class labels [28], in clustering [29], and to rank multi-cluster structure [30].

### 3.2. Laplacian Scores to Rank Features for Unsupervised Learning

To reduce the volume of data for specific tasks, class labels are normally available for supervised learning. However in many applications, feature reduction is needed for general usage, not limited to a specific task. This falls into unsupervised learning. Laplacian scores can rank features and users can select important features from the resulting rank [25] for the situations where no class label is available.

The similarity $S_{i,j}$ is defined as:

(2) $$S_{i,j}=exp\left(-\left(\frac{D_{i,j}}{\delta }\right)\right)$$

where δ is a scale factor and $D_{i,j}$ is the distance of two data points *i* and *j* in a local neighborhood.

The $i^{th}$ element, Dg, of the Degree matrix *D* is defined as

(3) $$D_{g}\left(i,i\right)=\sum_{j=1}^{n}S_{i,j}$$

The Laplacian matrix is defined as the difference between the degree matrix $D_{g}$ and the similarity matrix *S*:

(4) $$L=D_{g}-S$$

Alternatively, the feature selection results agree with to minimize the value:

(5) $$\frac{\sum_{i,j}{\left(x_{ir}-x_{jr}\right)}^{2}\times S_{i,j}}{Var(x_{r})}$$

where *r* is the *r*th feature, $x_{ir}$ is the *i*th observation of the *r*th feature. This means that features with large variance is preferred.

In the next section, a simple IoT system is designed to install four sensor measurements (temp, humidity, lighting, soil moisture) to monitor an environment. Then our planned feature reduction will be tested IoT systems. We will select more important features from the aforementioned four and evaluate whether the reduced dataset can achieve comparative performance with the full dataset.

## 4. Problem Definition and System Architecture

This section starts with the design of the IoT system architecture, followed by five building blocks and their choices of hardware/software for implementation. The gathered data of the real-world plant-monitoring IoT system is then used to test the proposed data-mining method.

### 4.1. System Architecture

The proposed system will be able to (1) collect data from sensors to monitoring agriculture related variables; (2) transmit such data to the gateway; (3) facilitate the gateway to send data to the cloud server; (4) enable data to be displayed at mobile APP or a client service.

**Figure 1:**
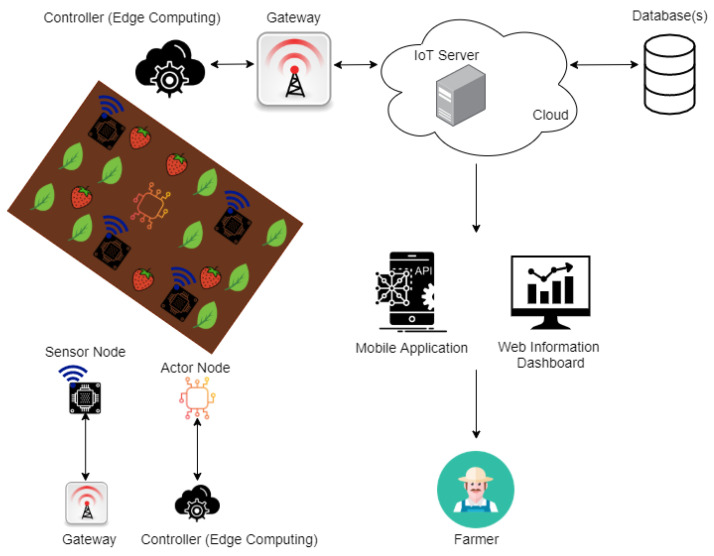
Overview of IoT system for strawberry-plant monitoring using LoRaWAN.

The overall system design is illustrated in Figure 1. Starting from the left, sensors/actuators for monitoring, such as temperature, humidity, light intensity and soil moisture are attached to a low-cost development platform. This platform consists of both a FRDM-K64F ARM mbed evaluation board (as the base) and a SX1272MB2xAS LoRa radio shield, to be explained later in this section. The main function of this platform is to transmit sensor data to a gateway. This cluster of physically connected devices is named “LoRa Node” in this paper. The LoRa Node sits next to the test site, for example, a plant.

The LoRa Node is transmitting data to a Gateway, using LoRa wireless communication. This wireless communication will be explained in Section 4.4. The Gateway is responsible for establishing an IP communication with, and sending data to an IoT Cloud Server. The Cloud Server sends data and its visualization to the end-user(s) through web and mobile dashboards. The following sections will explain the main building blocks in details.

### 4.2. IoT Platform Development

This platform consists of both a FRDM-K64F ARM mbed evaluation board (as the base) and a SX1272MB2xAS LoRa radio shield, to be explained later in this section. The main function of this platform is to transmit sensor data to a gateway. This cluster of physically connected devices is named “LoRa Node” in this paper. The LoRa Node sits next to the test site, for example, a plant.

The LoRa Node is transmitting data to a Gateway, using LoRa wireless communication. This wireless communication will be explained in Section 4.4. The Gateway is responsible for establishing an IP communication with, and sending data to an IoT Cloud Server. The Cloud Server sends data and its visualization to the end-user(s) through web and mobile dashboards. The following sections will explain the main building blocks in detail.

#### 4.2.1. Sensors

There is a rich range of sensors available in the market. The sensors chosen here are examples.

##### Soil Moisture Sensor

A soil moisture sensor detects the moisture of soil based on soil resistance measurement. In other words, sensor output value will decrease once soil moisture deficits. The output signal from the sensor is an analog value [31]. Notice that its measurements can be converted to a specific unit (e.g., voltage extraction) by employing FRDM–K64F ARM mbed board’s 16-bit ADC converter for meaningful data. The soil resistance measurement is in a range of 0 to 5 Volts soil moisture excitation. For instance, the soil resistance measurement can be calculated using the analog value as:

(6) moistureVoltage=moistureAnalog∗(5.0/65,536.0)

##### Temperature and Humidity Sensor

The chosen temperature and humidity sensor provides both temperature and humidity measurements as a pre-calibrated digital output using a negative temperature coefficient thermistor and a capacitive sensor element, accordingly [32]. Its detailed characteristics can be viewed through Table 1. At the beginning, the temperature and humidity sensor starts running the active mode from the low-power consumption mode once MCU sends a trigger signal. As a result, 40-bit data is collected back by the MCU consisting of 16-bit humidity data, 16-bit temperature data and 8-bit checksum number.

##### Light-Intensity Sensor

A light-intensity sensor exposes the intensity of light based on the resistance value of a photo-resistor (for the device chosen, GL5528 photo-resistor (Seeed, Shenzhen, China) ). In particular, the resistance of a photo-resistor increases when the intensity of light decreases. The output signal is an analog value [33]. The measurements can be converted to a specific unit (e.g., voltage extraction) by deploying FRDM–K64F ARM mbed board’s 16-bit ADC converter for meaningful data gathering. For example, the following calculation:

(7) lightVoltage=lightAnalog ∗ (5.0/65,536.0)

can be considered to be a 0 to 5 Volts light-intensity excitation.

#### 4.2.2. Lora Node Platform

As shown in Figure 1, a development platform attaches sensors and a transceiver send such data to a gateway.

##### FRDM–K64F ARM Mbed Board

FRDM–K64F ARM mbed board (ARM mbed, Cambridge, UK) is an ultra-low-cost development platform designed by NXP in collaboration with ARM mbed [34]. FRDM–K64F ARM mbed board will be the base device of LoRa Node along with SX1272MB2xAS LoRa shield and temperature, humidity, light intensity and soil moisture sensors. The sensors are physically attached on it. The specification of a FRDM–K64F ARM mbed board is in Table 2.

##### SX1272MB2xAS Semtech LoRa Shield

A SX1272MB2xAS Semtech LoRa shield (ARM mbed, Cambridge, UK) contains a SX1272 transceiver which features a spread communication using LoRa modulation over either 868 MHz or 915 MHz frequency [35]. For this particular product, the SX1272MB2xAS Semtech LoRa shield is attached to the base device FRDM–K64F ARM mbed board, constructing the desired LoRa node. The SX1272MB2xAS Semtech LoRa shield provides a reliable transmitting sensor measurement directly to a Gateway. The specification of the SX1272MB2xAS Semtech LoRa shield is in Table 3.

#### 4.2.3. Gateway

A Dragino LG01–P LoRa Gateway (Dragino, China) is a single-channel gateway that bridges the data gathered from the LoRa node (s) to the dedicated cloud service using either Wi-Fi, Ethernet, 3G or 4G cellular [36]. The specification of a Dragino LG01–P LoRa Gateway is in Table 4.

#### 4.2.4. Cloud Server

The “Things Network” Cloud Server is an open-source decentralized network service enabling devices (such as a LoRa Node) as well as Gateways (such as Dragino LG01-P LoRa Gateway) to be connected to it [37]. The Things Network is an open community with more than 3000 Gateways up and running, and 35,000 registered members. The goal of The Things Network is building a distributed IoT data infrastructure by creating sufficient data connectivity through LoRaWAN technology [37].

Certainly, there are various alternative options of Cloud Servers, such as the Mbed Cloud and the IBM Watson. Here the Things Network Cloud Server is chosen for its open-source providence and its concentration to the LoRaWAN technology. This aligns with this research which applies LoRaWAN into monitoring agriculture.

#### 4.2.5. Data Visualization and Client-Side Application

The “All Things Talk” application platform is chosen as it provides open-source data visualization through either web or mobile dashboards using an in-house HTTP API [38]. Some core features of All Things Talk API are real-time data gathering and instant notifications through either Web/Mobile dashboards or registered e-mail. Finally, All Things Talk API’s end-user(s) has/have the privilege of viewing, processing and downloading any historical measurements for data analysis purposes.

### 4.3. Software Development

#### Lora Node

Software architecture of LoRa Node can be observed in Algorithm 1. Functions, events and possible errors are illustrated. At first instance, setUp() function represents a local function call intended to initialize ARM mbed operating system environment as well as SX1272 Radio’s and IBM’s LMiC libraries configuration aspects. As a result, LMIC_setSession() application callback can then be implemented for acquiring an activation by personalization session. For a successful session establishment, static constants such as Network ID, Device Address, Network Session Key and Application Session Key extracted from The Things Network Cloud Server should be employed. After LMIC_setSession() callback, LoRa stack should output either EV_JOINED or EV_JOINED_FAILED event, indicating successful or unsuccessful join to the network service.

Then, getTemperatureHumidity() local function call is core for gathering related measurement parameters. Beyond that, DHT11 library which is intended to be used for temperature and humidity sensor’s implementation provides various error enumerations. Specifically, error enumerations of temperature and humidity sensors are ERROR_NONE, BUS_BUSY, ERROR_NOT_PRESENT, ERROR_ACK_TOO_LONG, ERROR_SYNC_TIMEOUT, ERROR_DATA_TIMEOUT, ERROR_CHECKSUM and ERROR_NO_PATIENCE in sequence. Additionally, both light-intensity and soil moisture measurement parameters are collected through getLightIntensity() and getSoilMoisture() local function calls, respectively.

Moving to data transmission, a time-triggered local function call should be initialized for sending desired LoRa packet to the Gateway in a context of set time interval. As with LMIC_setSession() application callback, events such as EV_TXCOMPLETE, EV_LOST_TSYNC or EV_LINK_DEAD should be outputted from transmit() function call indicating whether LoRa packet had successfully be transmitted to the connected Gateway. Apart from that, IBM’s LMiC library provides a _setTimedCallback() application callback which settles the program down until set time interval is being triggered signaling the next LoRa packet transmission.

Following embedded systems good principles, reset button deployment is giving the opportunity of completely resetting the LoRa Node, manually, at any time.

Finally, yet importantly, software architecture of LoRa Node has been implemented in a sequential form, avoiding any unnecessary computational complexity which could result in poor performance and increased power-consumption.
**Algorithm 1:** LoRa Node algorithmic software architecture.
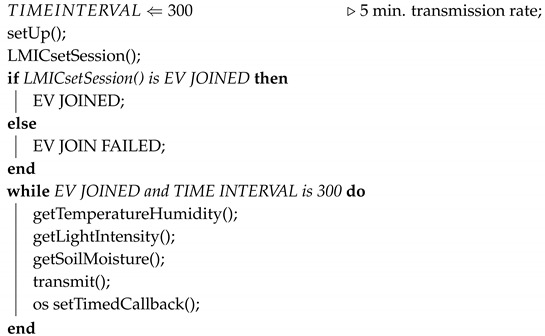


### 4.4. Network Architecture

This section provides the implementation of network architecture.

#### 4.4.1. Gateway

**Figure 2:**
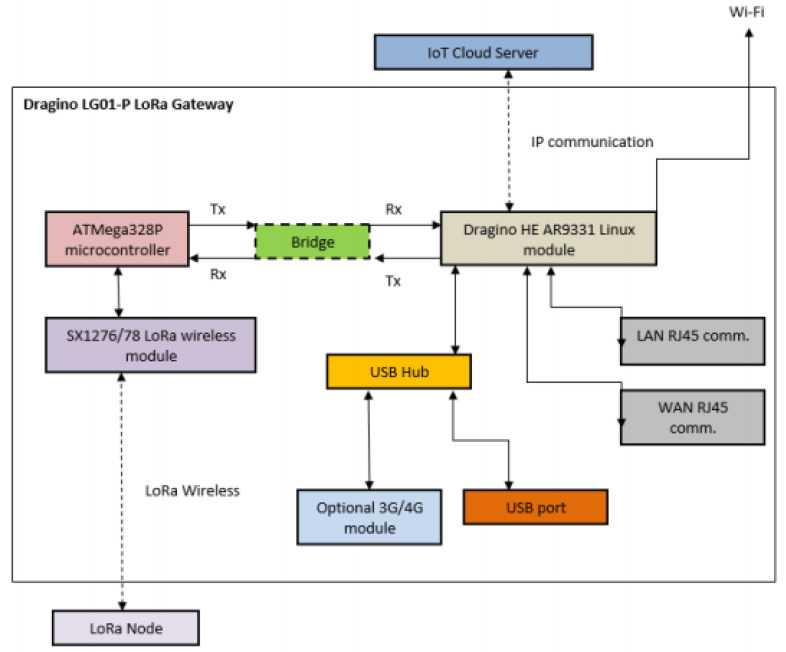
Block diagram of Dragino LG01–P LoRa Gateway architecture [36].

The LoRa Gateway’s block architecture is given in Figure 2. This Gateway can handle LoRa packets coming from the LoRa Node using the SX1276/78 LoRa wireless module which is attached on ATMega328P micro-controller. Then the Arduino environment communicates and passes LoRa packets to the Dragino HE AR9331 Linux module by employing a bridge library.

The Linux environment of Dragino’s LG01–P LoRa Gateway provides three different options for bridging the LoRa wireless network to an IP network for the successful transmission of LoRa packets to a Cloud Server: namely 802.11 b/g/n Wi-Fi, Ethernet (LAN and WAN RJ45 communications), and 3G/4G module. Please note that the chosen Dragino LG01–P LoRa Gateway does not include an internal 3G/4G module. As a result, cellular communications cannot be applied to our IoT system for agriculture.

The gateway is configured in a way that acts as the “middle station” between the LoRa Node and the IoT Cloud Server.

#### 4.4.2. Cloud Server

**Figure 3:**
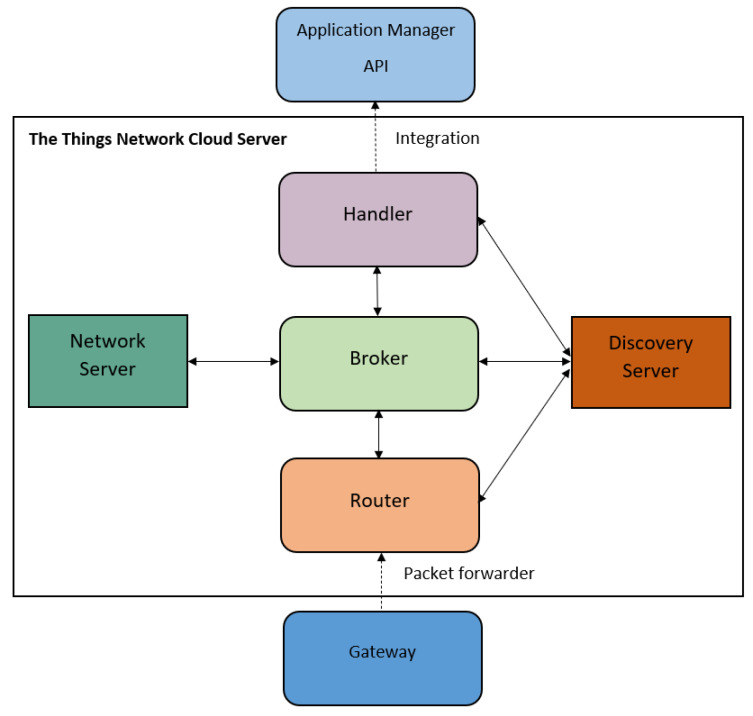
Block diagram of The Things Network Cloud Server architecture [39].

The block architecture of the “Things Network” Cloud Server is illustrated in Figure 3. Its open-sourced elements such as packet forwarder, router, broker, handler, network and discovery servers enable the employment of the LoRaWAN standard for IoT systems [39].

The main functionality of this cloud server includes: first, this cloud server forwards LoRa messages using a remotely configurable and secure packet forwarder [39]. Then, the router micro- service is liable for identifying a broker to forward the LoRa message [39]. When it comes to the handling procedure, a micro-service handler is reliable to encrypt as well as decrypt the play-load and therefore publishes it to the desired Application Manager API through a suitable integration [39]. Please note that the integration functionality bridges The Things Network Cloud Server with the IoT applications to support data visualization, analysis and storage [39].

In this infrastructure, both Discovery and Network servers are being employed. The discovery server keeps track of network’s components such as router, broker and handler. On the other hand, the network server monitors device states as well as device registries [39].

This cloud server is designed and implemented in a distributed and scalable way by allowing high-performance, high-availability and end-to-end security [39]. In addition, stack components such as gateway software, device libraries, cloud routing services and integration are being covered [39].

#### 4.4.3. Client/User Interface API

**Figure 4:**
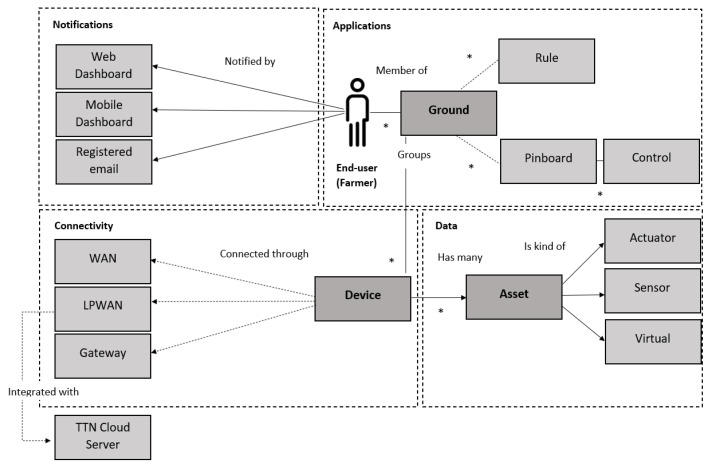
Domain diagram of All Things Talk API architecture [40].

We have chosen “All Things Talk API” to support clients and User Interface. Its architecture is illustrated in Figure 4. Entities such as applications, notifications, connectivity and data management, together build up an application manager API. This offers end users the opportunities to visualize, store and process gathered sensor data (measurements).

The All Things Talk API offers the choices of joining a device through either WAN, LPWAN or Gateway connections. In particular, the LoRa Node of our IoT system for agriculture, is integrated through the Low-Power Wide-Area Network (LPWAN) connection with The Things Network Cloud Server.

In the IoT system for agriculture, the measurement parameters such as temperature, humidity, light intensity and soil moisture will be displayed on the client side. In addition, a virtual watchdog has been initialized for monitoring any potential warnings or errors.

## 5. Experimental Results, Analysis and Discussion

### 5.1. Experimental Set-Up and Sensors Calibration

**Figure 5:**
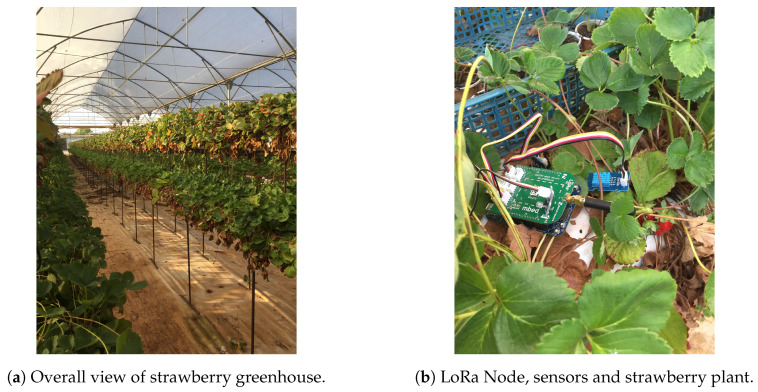
Greenhouse and LoRa node monitoring strawberry-plant growth.

To measure the functionality and performance of the proposed LoRaWAN empowered IoT architecture and implementation for agriculture, a testbed has been setup. An indoor greenhouse is used for this purpose, as seen in Figure 5a.

The hardware used are the Dragino LG01-P LoRa Gateway, FRDM–K64F ARM mbed board, LoRa shield, light-intensity sensor, soil moisture sensor, temperature and humidity sensor. A strawberry plant in this greenhouse is used for the tests which could be assumed to be representative of a plot in the greenhouse.

Sensors are attached to FRDM–K64F ARM mbed board and Semtech SX1272MB2xAS LoRa shield as seen in Figure 5b. The temperature and humidity sensors are connected through the D6 digital input port of the LoRa shield, while the soil moisture sensors are attached by employing the A3 analog input port. Similarly, the light-intensity sensor is used through A1 analog input port of the LoRa shield.

The Gateway is placed approximately 100 m away, due to the size of the greenhouse, from the above connected devices. The required Internet connection of Dragino LG01-P LoRa Gateway is established by deploying the WAN port of the device connected to an Ethernet admission. After that, the soil moisture sensor is placed inside the soil surrounding the strawberry plant, while the temperature, humidity and light-intensity sensors settle nearby, as seen in Figure 5b.

Data is flashed into the FRDM–K64F ARM mbed evaluation board’s micro-controller through the Mbed online compiler. The IoT system runs as an autonomous time-triggered program based on set transmit interval. Once data is collected, it will be sent to the cloud server, i.e., “The Things Network” and consecutively to the client interface API, i.e., “All Things Talk API”.

**Figure 6:**
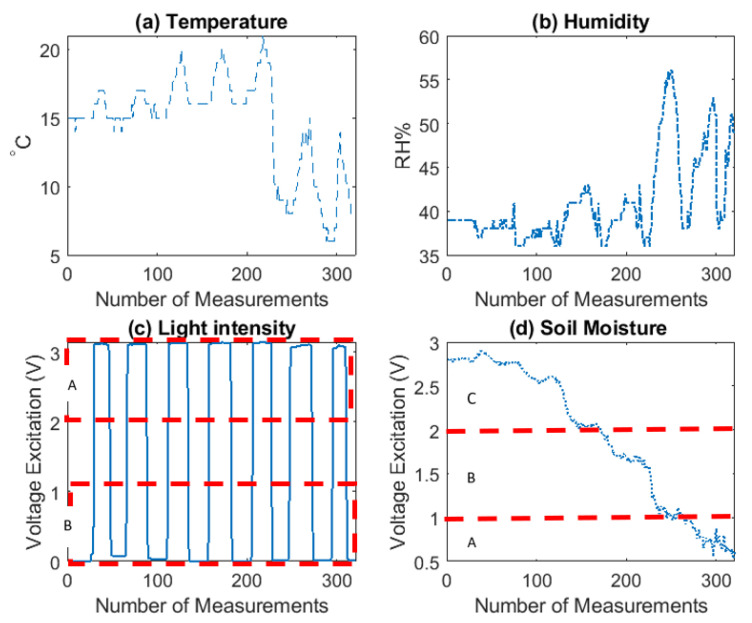
Visualization of sensors calibration test data. x axis is time (Number of Measurements). y axis represents the sensor readings. (**a**) Temp unit is °C, (**b**) Hum unit is % RH, (**c**) LightInt unit is Volts and (**d**) SoilMoist unit is Volts. Soil moisture calibrated against three different levels; (A) sensor in dry soil, (B) sensor in humid soil and (C) sensor in water. For the light-intensity sensor, calibration has been deployed for two different levels of light; (A) HIGH when sensor in daylight and (B) LOW when sensor in dark.

Before powering-up the whole IoT system, where compulsory, calibration tests have been conducted to measure the accuracy and stability of the sensor readings. For example, the temperature and humidity sensor is pre- calibrated with minimal sensitivity levels of humidity 1% RH and temperature 1 °C (see Table 1). On the other hand, for the soil moisture sensor, calibration has been conducted for three different levels of moisture; (A) sensor in dry soil, (B) sensor in humid soil and (C) sensor in water. Similarly, for the light-intensity sensor, calibration has been deployed for two different levels of light; (A) HIGH when sensor in daylight and (B) LOW when sensor in dark. The results for soil moisture and light-intensity sensors during calibration test are shown in Figure 6. Data gathered from temperature and humidity sensor is also being visualized for a more comprehensive review.

After the sensor calibration test, the real-environment test has been deployed. *Test 1 (Real-condition)* was set to transmit all sensor data at the interval of 300,000 milliseconds, which is 5 min.

### 5.2. Traffic Analysis

As seen in Table 5 and Table 6, Sensors Calibration Test is executed 4% data transmission loss, while Test 1 (Real-condition) is executed with 12% data transmission loss. It is clear that a higher number of measurements causes a higher data transmission loss.

### 5.3. Data Visualization

**Figure 7:**
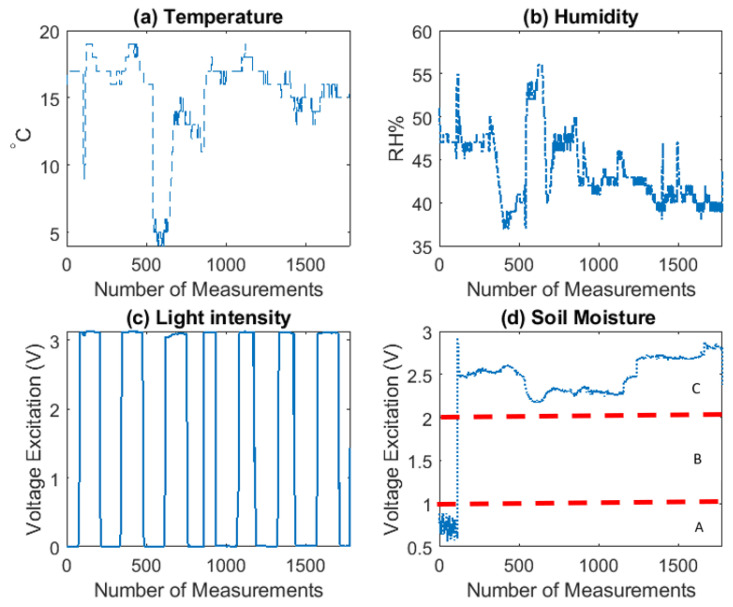
Visualization of Test 1 (Real-condition) data. x axis is time (Number of Measurements). y axis represents the sensor readings. (**a**) Temp unit is °C, (**b**) Hum unit is % RH, (**c**) LightInt unit is Volts and (**d**) SoilMoist unit is Volts. For decision-making purposes, three different watering events have been tested and can be observed; (A) Strawberry plant not watered, (B) Strawberry plant in humid soil and (C) Strawberry plant watered.

Figure 7 visualizes the sensing reading of temperature (legend: Temp), humidity (legend: Hum), light intensity (legend:LightInt) and soil moisture (legend: SoilMoist), collected in the Test 1 (Real-condition), in total of 1776 observations. For decision-making purposes, three different watering events have been tested and can be observed; (A) Strawberry plant not watered, (B) Strawberry plant in humid soil and (C) Strawberry plant watered.

#### Correlation Coefficients

Correlation coefficients are used to measure the dependence of the readings between two sensors *X* and *Y*. The Pearson correlation coefficient is defined as:

$$\rho \left(X,Y\right)=\frac{cov(X,Y)}{\sigma_{X}\sigma_{Y}},$$

where cov(X,Y) is the covariance of X and Y, and $\sigma_{X}$ and $\sigma_{Y}$ are the standard deviation of *X* and *Y*, respectively. The values of the coefficients can range from −1 to 1. Value −1 represents a directly negative correlation, 0 represents no correlation, and 1 represents a directly positive correlation.

Table 7 lists the ρ(X,Y) values for each pairwise variable combinations of temperature, humidity, light intensity and soil moisture, shorted as Temp, Hum, LightInt and SoilMoist respectively. It shows that Temp and Hum has a strong negative linear relationship, Temp and SoilMoist has a moderate positive linear relationship, and Hum and SoilMoist has a moderate negative linear relationship.

These findings are consistent with domain knowledge in agriculture: relative humidity relies on both pressure and temperature. At a lower temperature, less water vapor is needed to reach a high level of humidity. However, at a higher temperature, a higher water vapor is needed to obtain a high level of relative humidity.

### 5.4. Feature Selection and Evaluation

Data generating in this IoT system comes from four sensors. They measure temperature, humidity, light intensity and soil moisture. In the dataset, one feature contains the readings from one sensor. Data of each feature is being generated by the according sensor node. Laplacian scores are calculated to measure the important of features.

Laplacian scores here are for unsupervised learning. To further evaluate it, we test the result on the following example, as an application in future decision support.

#### Example in Decision Support

The outputs from the unsupervised method Laplacian scores can be used to for decision-making. For example, an expert labeled the data collected and decided when watering is needed. We compare the classification outcomes of using the selected features from using Laplacian scores and of using the all sensor data. Please note that the class label is only for one action here, while Laplacian scores is generated without class labels for general purpose.

Classifiers’ accuracy and performance measured using data inputs with 5 min transmission rate and last 2 h average. In both cases, classification conducted using the 4 and 3 most important features based on their scores.

The accuracy and performance of resulting classifiers using data inputs with 5 min transmission rate is shown in Table 8 for the 4 and 3 most important features, respectively.

The accuracy and performance of resulting classifiers using data inputs with last 2 h average is shown in Table 9 for the 4 and 3 most important features, respectively.

Overall, the classification results showed that the decision of watering or not a plant can be made using a reduced number of sensors. With 5 min transmission rate, the accuracy for decision-making achieved 95% when the least important feature has been removed. With the last 2 h average data set, the accuracy for decision-making achieved 97% when reducing the features to 3.

Often the acceptable level of accuracy is user defined, depending the nature of the subject or scenarios [41]. In this case, the accuracy reduces from nearly 100% to 97% and 95%, which means the error is within 5%. In statistics, when the type of error rate is within 5%, which is acceptable to have a 5% probability of incorrectly rejecting the true null hypothesis [41]. In addition, it is a common practice.

This approach can be promising for a large-scale deployment. The sum of a large amount of data from the least important sensor(s) might be reduced, if using appropriate data-mining methods to select sensors which are more important to the chosen decision-making.

## 6. Conclusions

This paper addresses the open challenge of feature reduction in IoT systems for agricultural plant-monitoring and decision-making support. Our data reduction approach is unsupervised learning using Laplacian scores. This approach is especially useful when class labels are unavailable. Using similarity and difference, features are ranked, so that users can select the most important features, rather than the whole feature set. Giving high resolutions of some features in real-world IoT applications, this will help reduce the volume of data to be transmitted. To evaluate our proposal, a real-world strawberry-plant monitoring IoT system has been implemented, calibrated and tested, measuring real-condition parameters such as temperature, relative humidity, soil moisture and light intensity. Our research has demonstrated that the proposed feature reduction can significantly reduce the volume data required to be transferred from the LoRa Node (edge device) to the network, while keeping the IoT system functioning at high accuracy levels. Moreover, the proposed IoT system has been tested on a specific decision-making support task (to water or not to water). The experimental results clearly show that the accuracy of decision-making on the reduced data decreases at an acceptable level (only 3–5%). The proposed research can potentially be used and provide insights for a rich range of decision-making tasks related to agricultural monitoring which can release the burden of data volume off the IoT systems.

In the future, this work can be expanded to another decision-making task except for watering a plant. For instance, if a greenhouse includes cooling fans, the event of turning them on/off could be controlled through an IoT system, similarly to what is proposed above. Strawberry and any other plants are very sensitive to very high/low level values of temperature or relative humidity so this could prevent them from being destroyed. Moreover, farmers can take advantage of this decision-making support to become more efficient on the usage of cooling fans, preventing high amount electricity bills. This decision-making scenario is planned to be conducted in the future when a greenhouse with such cooler fans is identified.

## Figures and Tables

**Table 1:** Temperature and Humidity Sensor Main Characteristics.

Grove Temperature and Humidity Sensor	
VCC	3.3–5 Volts
Measuring Range: Temperature	0–50 °C
Measuring Range: Humidity	20–90%
Sensitivity: Humidity	1%
Sensitivity: Temperature	1 °C

**Table 2:** FRDM–K64F ARM mbed board Main Hardware Specifications.

FRDM–K64F ARM Mbed Board	
MCU	Kinetis MK64FN1M0VLL12 (ARM Cortex-M4)
Flash	1024 KB
RAM	256 KB
CPU max. frequency	120 MHz

**Table 3:** SX1272MB2xAS Semtech LoRa shield Main Hardware Specifications.

SX1272MB2xAS Semtech LoRa Shield	
Transceiver	SX1272
Frequency Ranges	868 MHz and 915 MHz
Link Budget	157dB max.
Sensitivity	down to –137 dBm
Bit-Rate	300 kbps
Dynamic Range RSSI	127 dB

**Table 4:** Dragino LG01–P LoRa Gateway Main Hardware Specifications.

Dragino LG01–P LoRa Gateway	
Processor	400 MHz
MCU	ATMega328P
Flash	32 KB
Link Budget	168dB max.
Dynamic Range RSSI	127 dB
Bit-Rate	up to 300 kbps
RJ45 Ports	2 (WAN and LAN)
Wi-Fi	IEEE 802.11 b/g/n
Power Input	12V DC

**Table 5:** Sensors Calibration Test Traffic Analysis.

Sensors Calibration Test Traffic Analysis (321 Num. of Measurements).	
LoRa packets to send	336
LoRa packets to arrive	321
LoRa packets lost	15
LoRa packet loss percentage	4%

**Table 6:** Test 1 (Real-condition) Traffic Analysis.

Test 1 (Real-Condition) Traffic Analysis (1776 Num. of Measurements).	
LoRa packets to send	2016
LoRa packets to arrive	1776
LoRa packets lost	240
LoRa packet loss percentage	12%

**Table 7:** Correlation coefficients ρ values.

ρ	Temp	Hum	LightInt	SoilMoist
Temp	1	−0.8381	0.34	0.6573
Hum		1	−0.2273	−0.685
LightInt			1	0.0148
SoilMoist				1

**Table 8:** The accuracy and performance of resulting classifiers using data inputs with 5 min transmission rate for the 4 and 3 most important features.

Features	Correctly Classified Instances	Incorrectly Classified Instances
Hum, Temp, Light, Soil	1776 (100%)	0 (0%)
Hum, Temp, Light	1680 (94.5946%)	96 (5.4054%)

**Table 9:** The accuracy and performance of resulting classifiers using data inputs with last 2 h average for the 4 and 3 most important features.

Features	Correctly Classified Instances	Incorrectly Classified Instances
Hum, Temp, Light, Soil	1752 (99.943%)	1 (0.057%)
Hum, Temp, Light	1698 (96.8625%)	55 (3.1375%)
